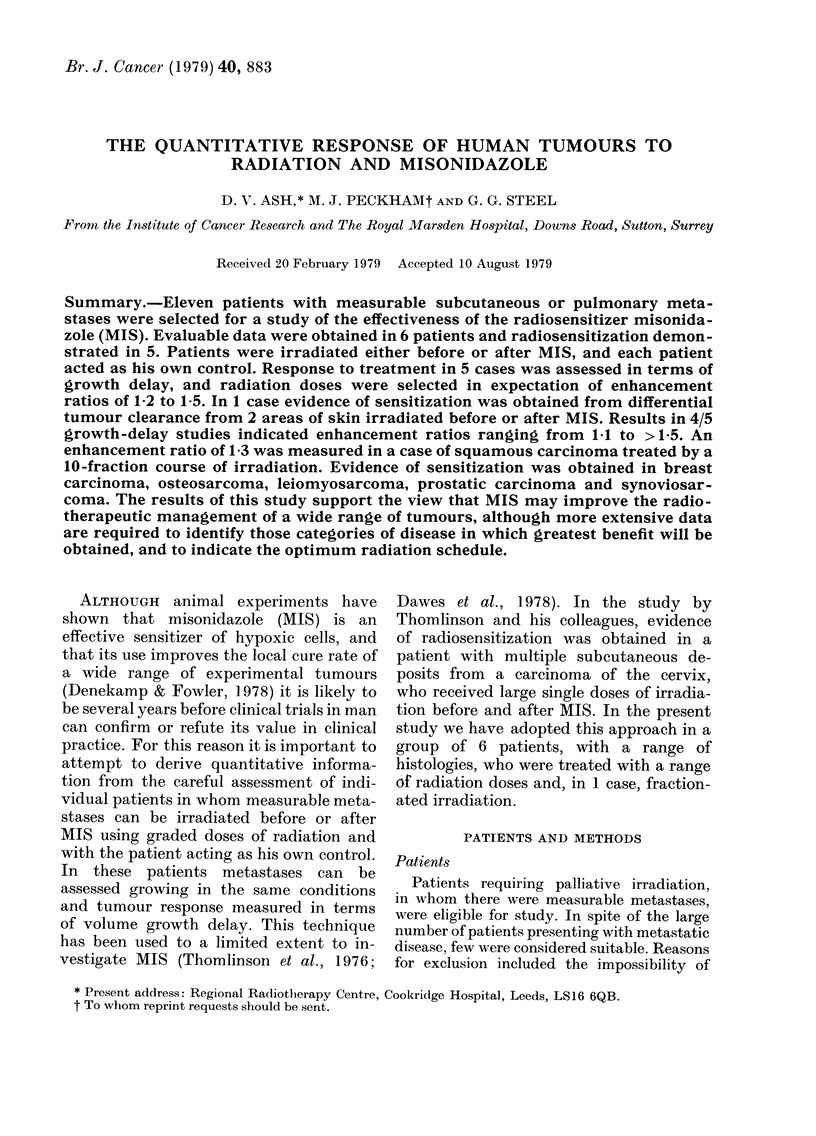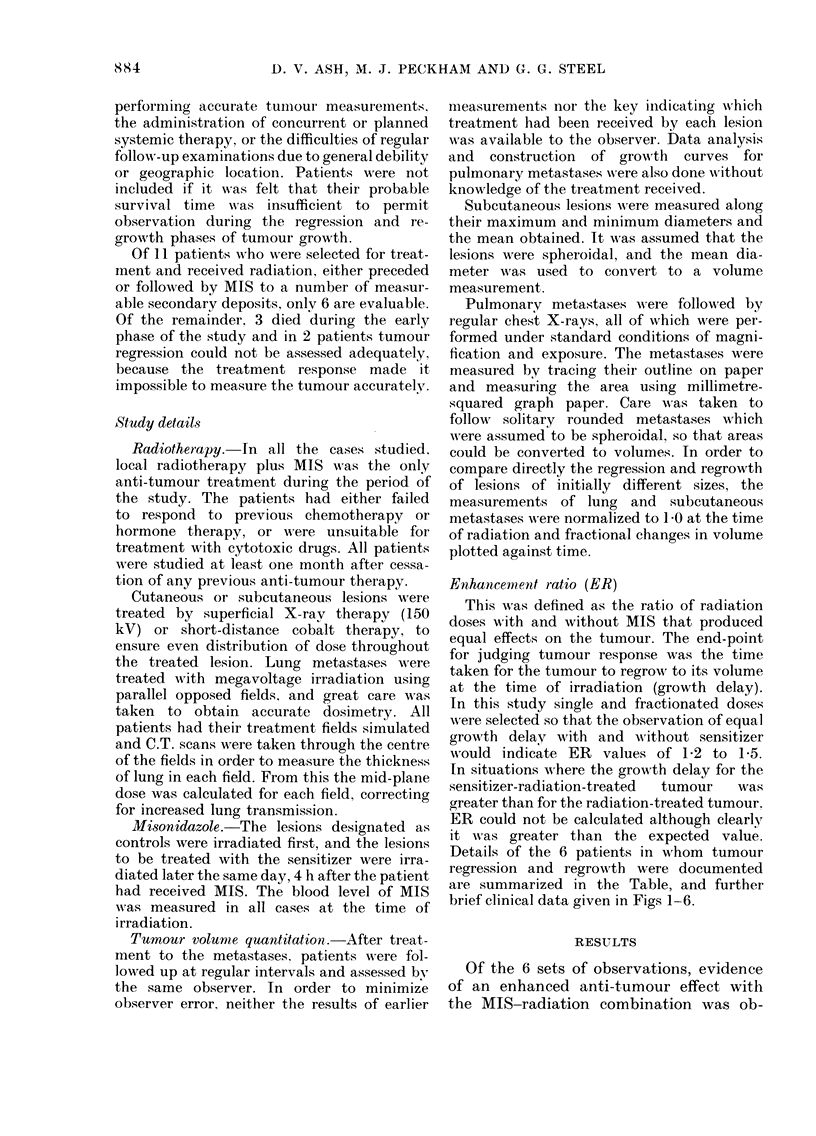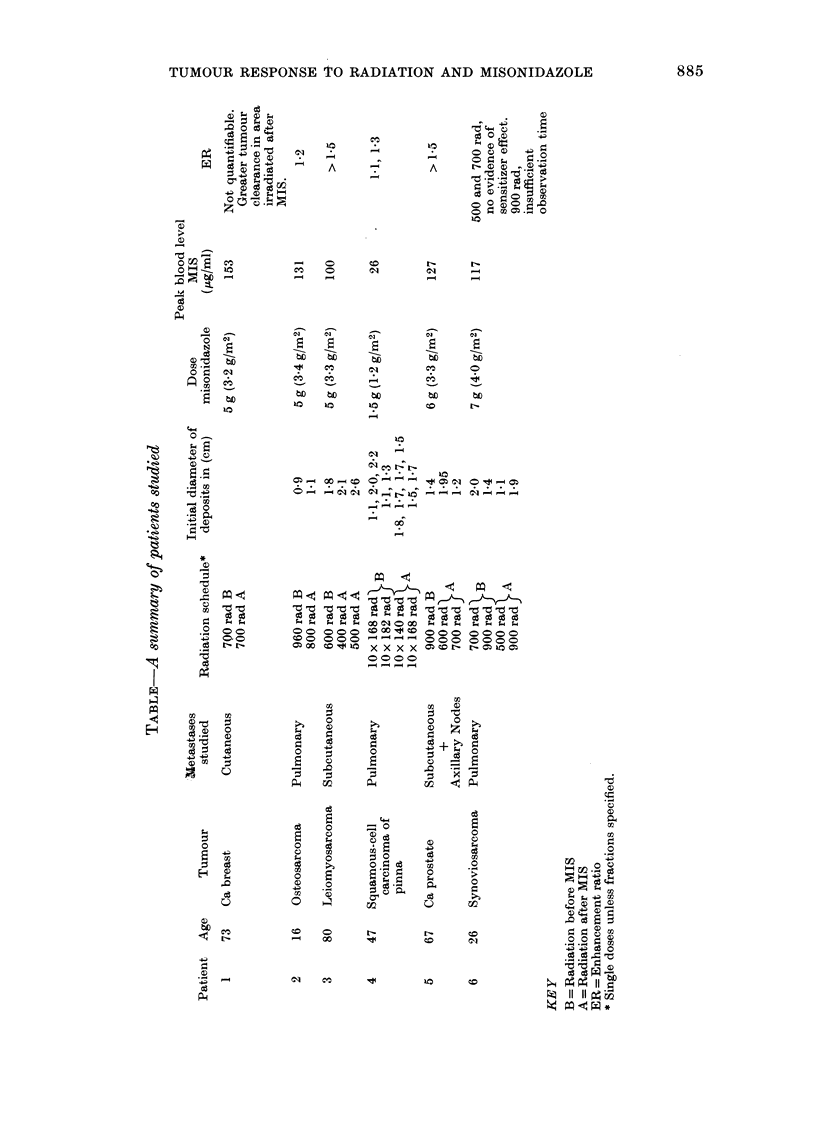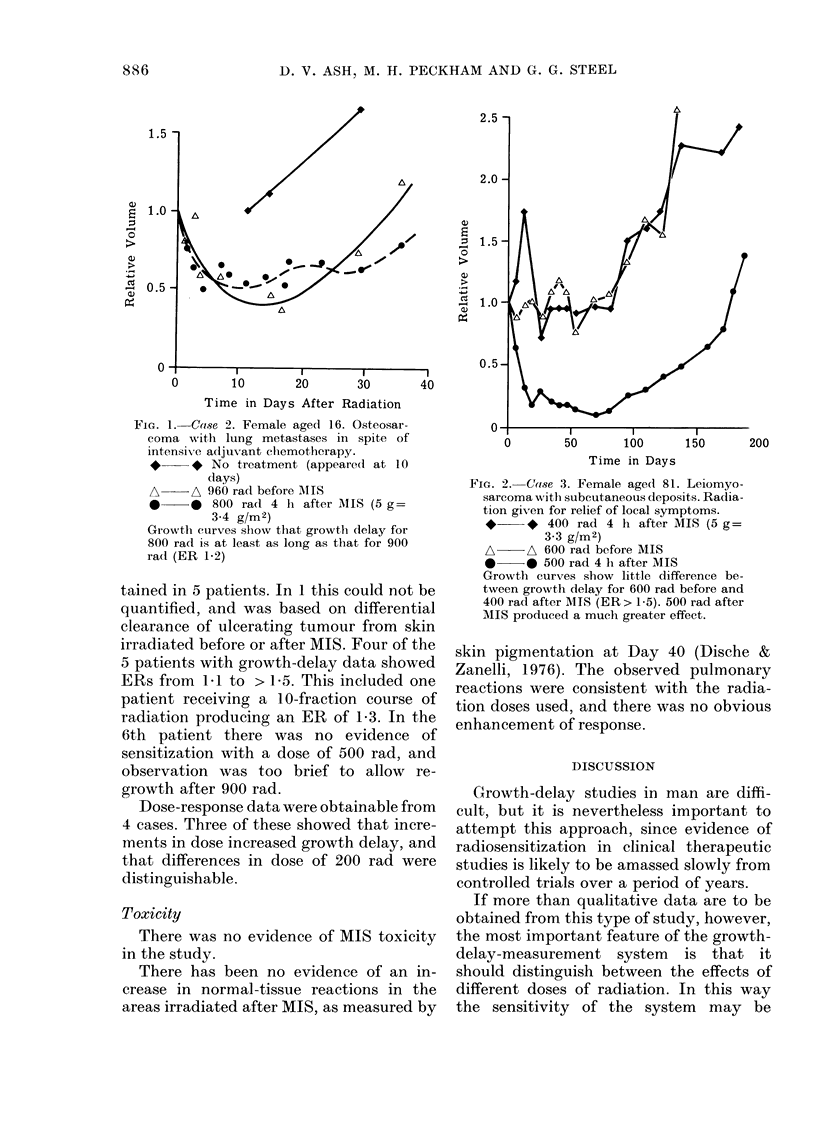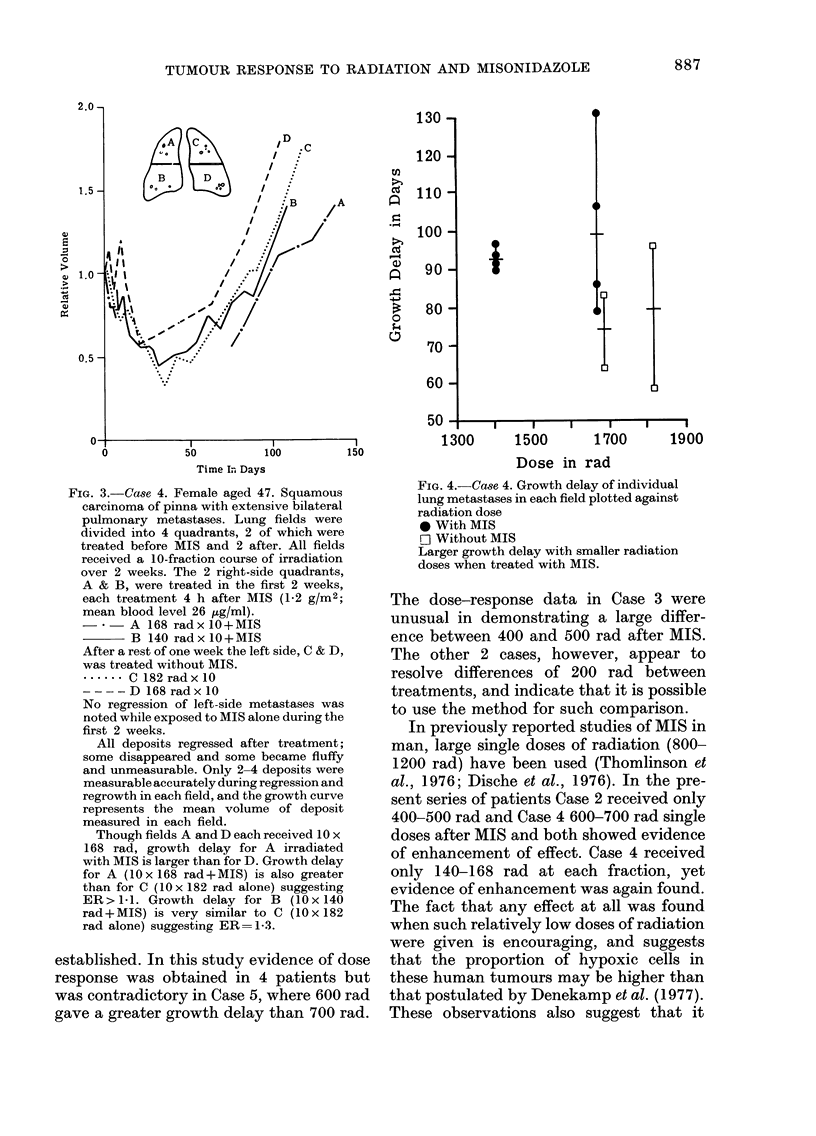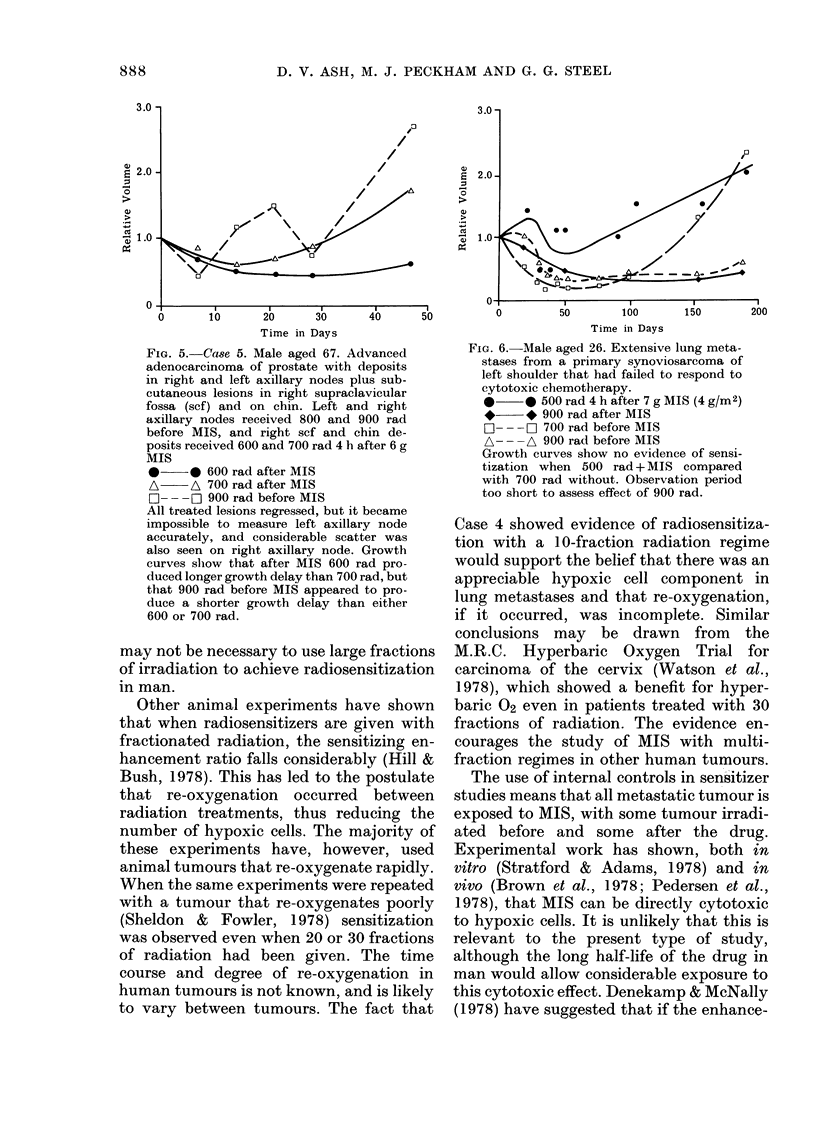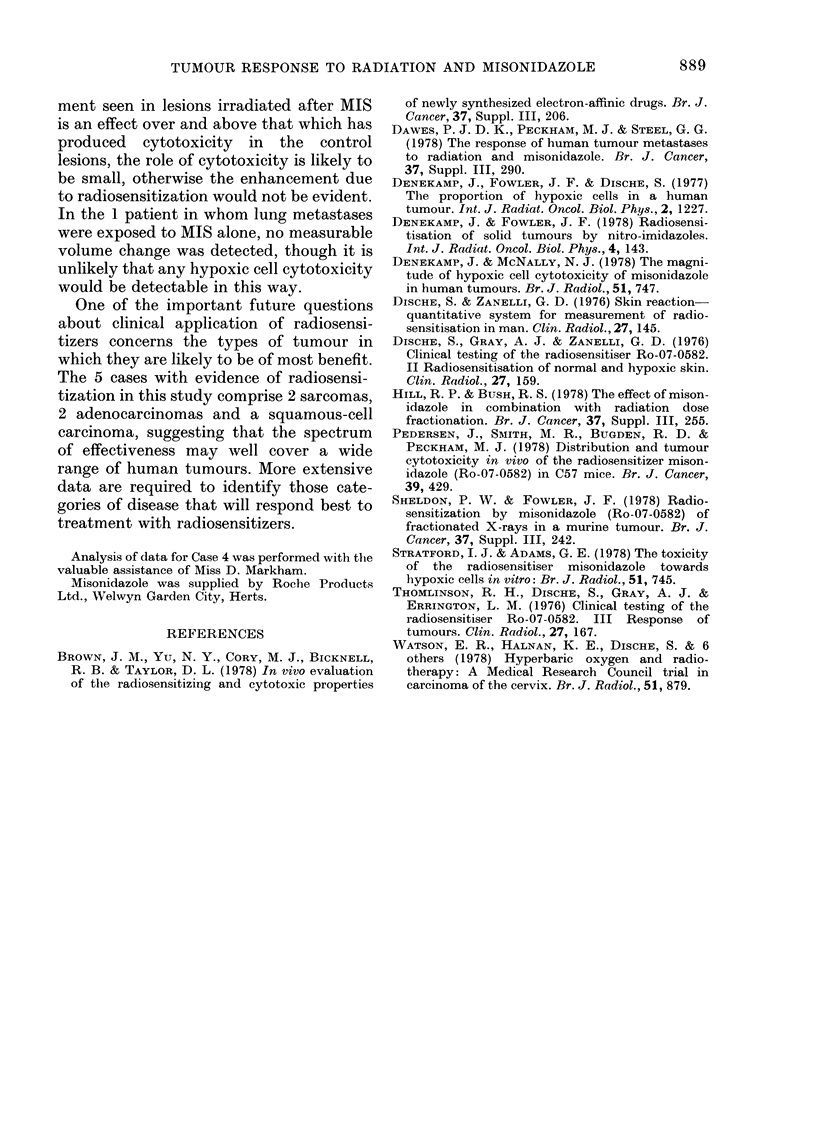# The quantitative response of human tumours to radiation and misonidazole.

**DOI:** 10.1038/bjc.1979.281

**Published:** 1979-12

**Authors:** D. V. Ash, M. J. Peckham, G. G. Steel

## Abstract

Eleven patients with measurable subcutaneous or pulmonary metastases were selected for a study of the effectiveness of the radiosensitizer misonidazole (MIS). Evaluable data were obtained in 6 patients and radiosensitization demonstrated in 5. Patients were irradiated either before or after MIS, and each patient acted as his own control. Response to treatment in 5 cases was assessed in terms of growth delay, and radiation doses were selected in expectation of enhancement ratios of 1.2 to 1.5. In 1 case evidence of sensitization was obtained from differential tumour clearance from 2 areas of skin irradiated before or after MIS. Results in 4/5 growth-delay studies indicated enhancement ratios ranging from 1.1 to greater than 1.5. An enhancement ratio of 1.3 was measured in a case of squamous carcinoma treated by a 10-fraction course of irradiation. Evidence of sensitization was obtained in breast carcinoma, osteosarcoma, leiomyosarcoma, prostatic carcinoma and synoviosarcoma. The results of this study support the view that MIS may improve the radiotherapeutic management of a wide range of tumours, although more extensive data are required to identify those categories of disease in which greatest benefit will be obtained, and to indicate the optimum radiation schedule.


*Br. J. Cancer* (1979) **40**, 883

### THE QUANTITATIVE RESPONSE OF HUMAN TUMOURS TO RADIATION AND MISONIDAZOLE

D. V. ASH,* M. J. PECKHAM† AND G. G. STEEL

*From the Institute of Cancer Research and The Royal Marsden Hospital, Downs Road, Sutton, Surrey*

Received 20 February 1979 Accepted 10 August 1979

**Summary.—Eleven patients with measurable subcutaneous or pulmonary metastases were selected for a study of the effectiveness of the radiosensitizer misonidazole (MIS). Evaluable data were obtained in 6 patients and radiosensitization demonstrated in 5. Patients were irradiated either before or after MIS, and each patient acted as his own control. Response to treatment in 5 cases was assessed in terms of growth delay, and radiation doses were selected in expectation of enhancement ratios of 1·2 to 1·5. In 1 case evidence of sensitization was obtained from differential tumour clearance from 2 areas of skin irradiated before or after MIS. Results in 4/5 growth-delay studies indicated enhancement ratios ranging from 1·1 to >1·5. An enhancement ratio of 1·3 was measured in a case of squamous carcinoma treated by a 10-fraction course of irradiation. Evidence of sensitization was obtained in breast carcinoma, osteosarcoma, leiomyosarcoma, prostatic carcinoma and synoviosarcoma. The results of this study support the view that MIS may improve the radiotherapeutic management of a wide range of tumours, although more extensive data are required to identify those categories of disease in which greatest benefit will be obtained, and to indicate the optimum radiation schedule.**

ALTHOUGH animal experiments have shown that misonidazole (MIS) is an effective sensitizer of hypoxic cells, and that its use improves the local cure rate of a wide range of experimental tumours (Denekamp & Fowler, 1978) it is likely to be several years before clinical trials in man can confirm or refute its value in clinical practice. For this reason it is important to attempt to derive quantitative information from the careful assessment of individual patients in whom measurable metastases can be irradiated before or after MIS using graded doses of radiation and with the patient acting as his own control. In these patients metastases can be assessed growing in the same conditions and tumour response measured in terms of volume growth delay. This technique has been used to a limited extent to investigate MIS (Thomlinson *et al.*, 1976; Dawes *et al.*, 1978). In the study by Thomlinson and his colleagues, evidence of radiosensitization was obtained in a patient with multiple subcutaneous deposits from a carcinoma of the cervix, who received large single doses of irradiation before and after MIS. In the present study we have adopted this approach in a group of 6 patients, with a range of histologies, who were treated with a range of radiation doses and, in 1 case, fractionated irradiation.

### PATIENTS AND METHODS

### *Patients*

Patients requiring palliative irradiation, in whom there were measurable metastases, were eligible for study. In spite of the large number of patients presenting with metastatic disease, few were considered suitable. Reasons for exclusion included the impossibility of

* Present address: Regional Radiotherapy Centre, Cookridge Hospital, Leeds, LS16 6QB.
† To whom reprint requests should be sent.

performing accurate tumour measurements, the administration of concurrent or planned systemic therapy, or the difficulties of regular follow-up examinations due to general debility or geographic location. Patients were not included if it was felt that their probable survival time was insufficient to permit observation during the regression and regrowth phases of tumour growth.

Of 11 patients who were selected for treatment and received radiation, either preceded or followed by MIS to a number of measurable secondary deposits, only 6 are evaluable. Of the remainder, 3 died during the early phase of the study and in 2 patients tumour regression could not be assessed adequately, because the treatment response made it impossible to measure the tumour accurately.

### *Study details*

*Radiotherapy.*—In all the cases studied, local radiotherapy plus MIS was the only anti-tumour treatment during the period of the study. The patients had either failed to respond to previous chemotherapy or hormone therapy, or were unsuitable for treatment with cytotoxic drugs. All patients were studied at least one month after cessation of any previous anti-tumour therapy.

Cutaneous or subcutaneous lesions were treated by superficial X-ray therapy (150 kV) or short-distance cobalt therapy, to ensure even distribution of dose throughout the treated lesion. Lung metastases were treated with megavoltage irradiation using parallel opposed fields, and great care was taken to obtain accurate dosimetry. All patients had their treatment fields simulated and C.T. scans were taken through the centre of the fields in order to measure the thickness of lung in each field. From this the mid-plane dose was calculated for each field, correcting for increased lung transmission.

*Misonidazole.*—The lesions designated as controls were irradiated first, and the lesions to be treated with the sensitizer were irradiated later the same day, 4 h after the patient had received MIS. The blood level of MIS was measured in all cases at the time of irradiation.

*Tumour volume quantitation.*—After treatment to the metastases, patients were followed up at regular intervals and assessed by the same observer. In order to minimize observer error, neither the results of earlier measurements nor the key indicating which treatment had been received by each lesion was available to the observer. Data analysis and construction of growth curves for pulmonary metastases were also done without knowledge of the treatment received.

Subcutaneous lesions were measured along their maximum and minimum diameters and the mean obtained. It was assumed that the lesions were spheroidal, and the mean diameter was used to convert to a volume measurement.

Pulmonary metastases were followed by regular chest X-rays, all of which were performed under standard conditions of magnification and exposure. The metastases were measured by tracing their outline on paper and measuring the area using millimetre-squared graph paper. Care was taken to follow solitary rounded metastases which were assumed to be spheroidal, so that areas could be converted to volumes. In order to compare directly the regression and regrowth of lesions of initially different sizes, the measurements of lung and subcutaneous metastases were normalized to 1·0 at the time of radiation and fractional changes in volume plotted against time.

### *Enhancement ratio (ER)*

This was defined as the ratio of radiation doses with and without MIS that produced equal effects on the tumour. The end-point for judging tumour response was the time taken for the tumour to regrow to its volume at the time of irradiation (growth delay). In this study single and fractionated doses were selected so that the observation of equal growth delay with and without sensitizer would indicate ER values of 1·2 to 1·5. In situations where the growth delay for the sensitizer-radiation-treated tumour was greater than for the radiation-treated tumour, ER could not be calculated although clearly it was greater than the expected value. Details of the 6 patients in whom tumour regression and regrowth were documented are summarized in the Table, and further brief clinical data given in Figs 1–6.

### RESULTS

Of the 6 sets of observations, evidence of an enhanced anti-tumour effect with the MIS–radiation combination was ob-

TABLE—*A summary of patients studied*

| Patient | Age | Tumour | Metastases studied | Radiation schedule* | Initial diameter of deposits in (cm) | Dose misonidazole | Peak blood level MIS ($\mu$g/ml) | ER |
|---|---|---|---|---|---|---|---|---|
| 1 | 73 | Ca breast | Cutaneous | 700 rad B<br>700 rad A | | 5 g (3·2 g/m²) | 153 | Not quantifiable. Greater tumour clearance in area irradiated after MIS. |
| 2 | 16 | Osteosarcoma | Pulmonary | 960 rad B<br>800 rad A | 0·9<br>1·1 | 5 g (3·4 g/m²) | 131 | 1·2 |
| 3 | 80 | Leiomyosarcoma | Subcutaneous | 600 rad B<br>400 rad A<br>500 rad A | 1·8<br>2·1<br>2·6 | 5 g (3·3 g/m²) | 100 | > 1·5 |
| 4 | 47 | Squamous-cell carcinoma of pinna | Pulmonary | 10 × 168 rad } B<br>10 × 182 rad } B<br>10 × 140 rad } A<br>10 × 168 rad } A | 1·1, 2·0, 2·2<br>1·1, 1·3<br>1·8, 1·7, 1·7, 1·5<br>1·5, 1·7 | 1·5 g (1·2 g/m²) | 26 | 1·1, 1·3 |
| 5 | 67 | Ca prostate | Subcutaneous + Axillary Nodes | 900 rad B<br>600 rad } A<br>700 rad } A | 1·4<br>1·95<br>1·2 | 6 g (3·3 g/m²) | 127 | > 1·5 |
| 6 | 26 | Synoviosarcoma | Pulmonary | 700 rad } B<br>900 rad } B<br>500 rad } A<br>900 rad } A | 2·0<br>1·4<br>1·1<br>1·9 | 7 g (4·0 g/m²) | 117 | 500 and 700 rad, no evidence of sensitizer effect. 900 rad, insufficient observation time |

*KEY*

B = Radiation before MIS

A = Radiation after MIS

ER = Enhancement ratio

* Single doses unless fractions specified.

FIG. 1.—*Case* 2. Female aged 16. Osteosarcoma with lung metastases in spite of intensive adjuvant chemotherapy.

◆——◆ No treatment (appeared at 10 days)

△——△ 960 rad before MIS

●——● 800 rad 4 h after MIS (5 g = 3·4 g/m$^2$)

Growth curves show that growth delay for 800 rad is at least as long as that for 900 rad (ER 1·2)

FIG. 2.—*Case* 3. Female aged 81. Leiomyosarcoma with subcutaneous deposits. Radiation given for relief of local symptoms.
◆——◆ 400 rad 4 h after MIS (5 g = 3·3 g/m$^2$)
△——△ 600 rad before MIS
●——● 500 rad 4 h after MIS
Growth curves show little difference between growth delay for 600 rad before and 400 rad after MIS (ER > 1·5). 500 rad after MIS produced a much greater effect.

tained in 5 patients. In 1 this could not be quantified, and was based on differential clearance of ulcerating tumour from skin irradiated before or after MIS. Four of the 5 patients with growth-delay data showed ERs from 1·1 to > 1·5. This included one patient receiving a 10-fraction course of radiation producing an ER of 1·3. In the 6th patient there was no evidence of sensitization with a dose of 500 rad, and observation was too brief to allow regrowth after 900 rad.

Dose-response data were obtainable from 4 cases. Three of these showed that increments in dose increased growth delay, and that differences in dose of 200 rad were distinguishable.

### *Toxicity*

There was no evidence of MIS toxicity in the study.

There has been no evidence of an increase in normal-tissue reactions in the areas irradiated after MIS, as measured by skin pigmentation at Day 40 (Dische & Zanelli, 1976). The observed pulmonary reactions were consistent with the radiation doses used, and there was no obvious enhancement of response.

### DISCUSSION

Growth-delay studies in man are difficult, but it is nevertheless important to attempt this approach, since evidence of radiosensitization in clinical therapeutic studies is likely to be amassed slowly from controlled trials over a period of years.

If more than qualitative data are to be obtained from this type of study, however, the most important feature of the growth-delay-measurement system is that it should distinguish between the effects of different doses of radiation. In this way the sensitivity of the system may be

FIG. 3.—*Case* 4. Female aged 47. Squamous carcinoma of pinna with extensive bilateral pulmonary metastases. Lung fields were divided into 4 quadrants, 2 of which were treated before MIS and 2 after. All fields received a 10-fraction course of irradiation over 2 weeks. The 2 right-side quadrants, A & B, were treated in the first 2 weeks, each treatment 4 h after MIS (1·2 g/m²; mean blood level 26 μg/ml).
— • — A 168 rad × 10 + MIS
——— B 140 rad × 10 + MIS
After a rest of one week the left side, C & D, was treated without MIS.
······ C 182 rad × 10
– – – – D 168 rad × 10
No regression of left-side metastases was noted while exposed to MIS alone during the first 2 weeks.

All deposits regressed after treatment; some disappeared and some became fluffy and unmeasurable. Only 2–4 deposits were measurable accurately during regression and regrowth in each field, and the growth curve represents the mean volume of deposit measured in each field.

Though fields A and D each received 10 × 168 rad, growth delay for A irradiated with MIS is larger than for D. Growth delay for A (10 × 168 rad + MIS) is also greater than for C (10 × 182 rad alone) suggesting ER > 1·1. Growth delay for B (10 × 140 rad + MIS) is very similar to C (10 × 182 rad alone) suggesting ER = 1·3.

FIG. 4.—*Case* 4. Growth delay of individual lung metastases in each field plotted against radiation dose
● With MIS
□ Without MIS
Larger growth delay with smaller radiation doses when treated with MIS.

established. In this study evidence of dose response was obtained in 4 patients but was contradictory in Case 5, where 600 rad gave a greater growth delay than 700 rad.

The dose–response data in Case 3 were unusual in demonstrating a large difference between 400 and 500 rad after MIS. The other 2 cases, however, appear to resolve differences of 200 rad between treatments, and indicate that it is possible to use the method for such comparison.

In previously reported studies of MIS in man, large single doses of radiation (800–1200 rad) have been used (Thomlinson *et al.*, 1976; Dische *et al.*, 1976). In the present series of patients Case 2 received only 400–500 rad and Case 4 600–700 rad single doses after MIS and both showed evidence of enhancement of effect. Case 4 received only 140–168 rad at each fraction, yet evidence of enhancement was again found. The fact that any effect at all was found when such relatively low doses of radiation were given is encouraging, and suggests that the proportion of hypoxic cells in these human tumours may be higher than that postulated by Denekamp *et al.* (1977). These observations also suggest that it

FIG. 5.—*Case* 5. Male aged 67. Advanced adenocarcinoma of prostate with deposits in right and left axillary nodes plus subcutaneous lesions in right supraclavicular fossa (scf) and on chin. Left and right axillary nodes received 800 and 900 rad before MIS, and right scf and chin deposits received 600 and 700 rad 4 h after 6 g MIS

●——● 600 rad after MIS

△——△ 700 rad after MIS

□- - -□ 900 rad before MIS

All treated lesions regressed, but it became impossible to measure left axillary node accurately, and considerable scatter was also seen on right axillary node. Growth curves show that after MIS 600 rad produced longer growth delay than 700 rad, but that 900 rad before MIS appeared to produce a shorter growth delay than either 600 or 700 rad.

FIG. 6.—Male aged 26. Extensive lung metastases from a primary synoviosarcoma of left shoulder that had failed to respond to cytotoxic chemotherapy.

●——● 500 rad 4 h after 7 g MIS (4 g/m$^2$)

◆——◆ 900 rad after MIS

□- - -□ 700 rad before MIS

△- - -△ 900 rad before MIS

Growth curves show no evidence of sensitization when 500 rad+MIS compared with 700 rad without. Observation period too short to assess effect of 900 rad.

may not be necessary to use large fractions of irradiation to achieve radiosensitization in man.

Other animal experiments have shown that when radiosensitizers are given with fractionated radiation, the sensitizing enhancement ratio falls considerably (Hill & Bush, 1978). This has led to the postulate that re-oxygenation occurred between radiation treatments, thus reducing the number of hypoxic cells. The majority of these experiments have, however, used animal tumours that re-oxygenate rapidly. When the same experiments were repeated with a tumour that re-oxygenates poorly (Sheldon & Fowler, 1978) sensitization was observed even when 20 or 30 fractions of radiation had been given. The time course and degree of re-oxygenation in human tumours is not known, and is likely to vary between tumours. The fact that Case 4 showed evidence of radiosensitization with a 10-fraction radiation regime would support the belief that there was an appreciable hypoxic cell component in lung metastases and that re-oxygenation, if it occurred, was incomplete. Similar conclusions may be drawn from the M.R.C. Hyperbaric Oxygen Trial for carcinoma of the cervix (Watson *et al.*, 1978), which showed a benefit for hyperbaric $O_2$ even in patients treated with 30 fractions of radiation. The evidence encourages the study of MIS with multifraction regimes in other human tumours.

The use of internal controls in sensitizer studies means that all metastatic tumour is exposed to MIS, with some tumour irradiated before and some after the drug. Experimental work has shown, both *in vitro* (Stratford & Adams, 1978) and *in vivo* (Brown *et al.*, 1978; Pedersen *et al.*, 1978), that MIS can be directly cytotoxic to hypoxic cells. It is unlikely that this is relevant to the present type of study, although the long half-life of the drug in man would allow considerable exposure to this cytotoxic effect. Denekamp & McNally (1978) have suggested that if the enhancement seen in lesions irradiated after MIS is an effect over and above that which has produced cytotoxicity in the control lesions, the role of cytotoxicity is likely to be small, otherwise the enhancement due to radiosensitization would not be evident. In the 1 patient in whom lung metastases were exposed to MIS alone, no measurable volume change was detected, though it is unlikely that any hypoxic cell cytotoxicity would be detectable in this way.

One of the important future questions about clinical application of radiosensitizers concerns the types of tumour in which they are likely to be of most benefit. The 5 cases with evidence of radiosensitization in this study comprise 2 sarcomas, 2 adenocarcinomas and a squamous-cell carcinoma, suggesting that the spectrum of effectiveness may well cover a wide range of human tumours. More extensive data are required to identify those categories of disease that will respond best to treatment with radiosensitizers.

Analysis of data for Case 4 was performed with the valuable assistance of Miss D. Markham.

Misonidazole was supplied by Roche Products Ltd., Welwyn Garden City, Herts.

### REFERENCES

Brown, J. M., Yu, N. Y., Cory, M. J., Bicknell, R. B. & Taylor, D. L. (1978) *In vivo* evaluation of the radiosensitizing and cytotoxic properties of newly synthesized electron-affinic drugs. *Br. J. Cancer*, **37**, Suppl. III, 206.

Dawes, P. J. D. K., Peckham, M. J. & Steel, G. G. (1978) The response of human tumour metastases to radiation and misonidazole. *Br. J. Cancer*, **37**, Suppl. III, 290.

Denekamp, J., Fowler, J. F. & Dische, S. (1977) The proportion of hypoxic cells in a human tumour. *Int. J. Radiat. Oncol. Biol. Phys.*, **2**, 1227.

Denekamp, J. & Fowler, J. F. (1978) Radiosensitisation of solid tumours by nitro-imidazoles. *Int. J. Radiat. Oncol. Biol. Phys.*, **4**, 143.

Denekamp, J. & McNally, N. J. (1978) The magnitude of hypoxic cell cytotoxicity of misonidazole in human tumours. *Br. J. Radiol.*, **51**, 747.

Dische, S. & Zanelli, G. D. (1976) Skin reaction—quantitative system for measurement of radiosensitisation in man. *Clin. Radiol.*, **27**, 145.

Dische, S., Gray, A. J. & Zanelli, G. D. (1976) Clinical testing of the radiosensitiser Ro-07-0582. II Radiosensitisation of normal and hypoxic skin. *Clin. Radiol.*, **27**, 159.

Hill, R. P. & Bush, R. S. (1978) The effect of misonidazole in combination with radiation dose fractionation. *Br. J. Cancer*, **37**, Suppl. III, 255.

Pedersen, J., Smith, M. R., Bugden, R. D. & Peckham, M. J. (1978) Distribution and tumour cytotoxicity *in vivo* of the radiosensitizer misonidazole (Ro-07-0582) in C57 mice. *Br. J. Cancer*, **39**, 429.

Sheldon, P. W. & Fowler, J. F. (1978) Radiosensitization by misonidazole (Ro-07-0582) of fractionated X-rays in a murine tumour. *Br. J. Cancer*, **37**, Suppl. III, 242.

Stratford, I. J. & Adams, G. E. (1978) The toxicity of the radiosensitiser misonidazole towards hypoxic cells *in vitro*: *Br. J. Radiol.*, **51**, 745.

Thomlinson, R. H., Dische, S., Gray, A. J. & Errington, L. M. (1976) Clinical testing of the radiosensitiser Ro-07-0582. III Response of tumours. *Clin. Radiol.*, **27**, 167.

Watson, E. R., Halnan, K. E., Dische, S. & 6 others (1978) Hyperbaric oxygen and radiotherapy: A Medical Research Council trial in carcinoma of the cervix. *Br. J. Radiol.*, **51**, 879.